# Misshapen Disruption Cooperates with *Ras^V12^* to Drive Tumorigenesis

**DOI:** 10.3390/cells10040894

**Published:** 2021-04-14

**Authors:** Du Kong, Jin-Yu Lu, Xiaoqin Li, Sihua Zhao, Wenyan Xu, Jinan Fang, Xing Wang, Xianjue Ma

**Affiliations:** 1 School of Medicine, Zhejiang University, Hangzhou 310058, China; kongdu@westlake.edu.cn; 2 Key Laboratory of Growth Regulation and Translational Research of Zhejiang Province, School of Life Sciences, Westlake University, Hangzhou 310024, China; zhaosihua@westlake.edu.cn (S.Z.); FangJinan@westlake.edu.cn (J.F.); 3 Westlake Laboratory of Life Sciences and Biomedicine, Hangzhou 310024, China; xuwenyan@westlake.edu.cn; 4 Institute of Biology, Westlake Institute for Advanced Study, Hangzhou 310024, China; 5 Baylor College of Medicine, Hematology & Oncology, Houston, TX 77054, USA; jinyu.lu@gmail.com; 6 College of Resources and Environmental Sciences, China Agricultural University, Beijing 100193, China; bs20193030289@cau.edu.cn; 7 Beijing Key Laboratory of Biodiversity and Organic Farming, Beijing 100193, China; 8 Key Laboratory of Structural Biology of Zhejiang Province, School of Life Sciences, Westlake University, Hangzhou 310024, China

**Keywords:** *Ras*, Msn, Ft, Hippo, tumorigenesis, *Drosophila*

## Abstract

Although *RAS* family genes play essential roles in tumorigenesis, effective treatments targeting *RAS*-related tumors are lacking, partly because of an incomplete understanding of the complex signaling crosstalk within *RAS*-related tumors. Here, we performed a large-scale genetic screen in *Drosophila* eye imaginal discs and identified *Misshapen* (*Msn*) as a tumor suppressor that synergizes with oncogenic *Ras* (*Ras^V12^*) to induce c-Jun N-terminal kinase (JNK) activation and Hippo inactivation, then subsequently leads to tumor overgrowth and invasion. Moreover, ectopic Msn expression activates Hippo signaling pathway and suppresses Hippo signaling disruption-induced overgrowth. Importantly, we further found that Msn acts downstream of protocadherin Fat (Ft) to regulate Hippo signaling. Finally, we identified *msn* as a Yki/Sd target gene that regulates Hippo pathway in a negative feedback manner. Together, our findings identified Msn as a tumor suppressor and provide a novel insight into *RAS*-related tumorigenesis that may be relevant to human cancer biology.

## 1. Introduction

The *RAS* genes (*HRAS*, *NRAS*, and *KRAS*) identified in 1982 are the most frequently mutated oncogenes in various human cancers [1,2]. There are substantial experiments showing that mutated *RAS* is a critical cancer driver and anti-*RAS* therapy is expected to be a promising direction for cancer treatment [3,4]. However, despite decades of efforts and breakthroughs, effective therapies are still underdeveloped for *RAS*-related tumors [1,2,3,4], largely because of the complexity of signaling crosstalk and synergetic effects within *RAS*-related tumors.

A large-scale genetic screen is an effective unbiased method to systematically dissect the genetic bases in *RAS*-related tumors. Over the last few decades, *Drosophila* has been proven to be an excellent model organism for cancer research [5,6,7,8] and a variety of tumor models have been established in *Drosophila* [9,10,11,12]. Importantly, the genetic screens in *Drosophila* have identified that the disruption of cell polarity genes (*scrib*, *dlg*, *lgl*) collaborate with oncogenic *Ras* (*Ras^V12^*) or Notch to promote tumor overgrowth and invasion [12,13]. Thus, these established tumor models and genetic tools make it feasible to conduct large-scale genetic screens in *Drosophila* to dissect the mechanisms of *RAS*-related cooperative oncogenesis.

Both c-Jun N-terminal kinase (JNK) and Hippo signaling pathways have been established in tumorigenesis. JNK pathway activation cooperates with Ras signaling and promotes tumor overgrowth and invasion [14]. Simultaneously, blocked JNK activity could dramatically reduce tumor overgrowth and invasion in various *Drosophila* tumor models [15,16,17]. Hippo signaling pathway has an essential role in organ size control and cell proliferation; its dysregulation causes a lot of human disease, including cancer [18,19,20,21]. 

Recently, we performed a large-scale ethyl methanesulphonate (EMS)-induced genetic screen, aiming to uncover novel tumor suppressors that could synergistically promote *Ras^V12^*-induced tumor overgrowth [16,22]. Here, we identified that *misshapen* (*msn*, CG16973) acts as a tumor suppressor that cooperates with oncogenic *Ras^V12^* to significantly promote tumor overgrowth and invasion by simultaneously activating JNK pathway and inactivating Hippo pathway. We found that Msn overexpression impeded Hippo-disruption-induced overgrowth by genetically acting downstream of protocadherin Fat (Ft). Additionally, we revealed that *msn* is a potential Yki/Sd target gene and it regulates Hippo pathway in a negative feedback manner. Together, these findings not only uncovered Msn as a novel regulator of *RAS*-related cooperative oncogenesis, but also provided a potential therapeutic target for *RAS*-related tumors.

## 2. Results

### 2.1. msn Mutant Synergizes with Oncogenic Ras^V12^ to Drive Tumorigenesis and Invasion

**Figure 1:**
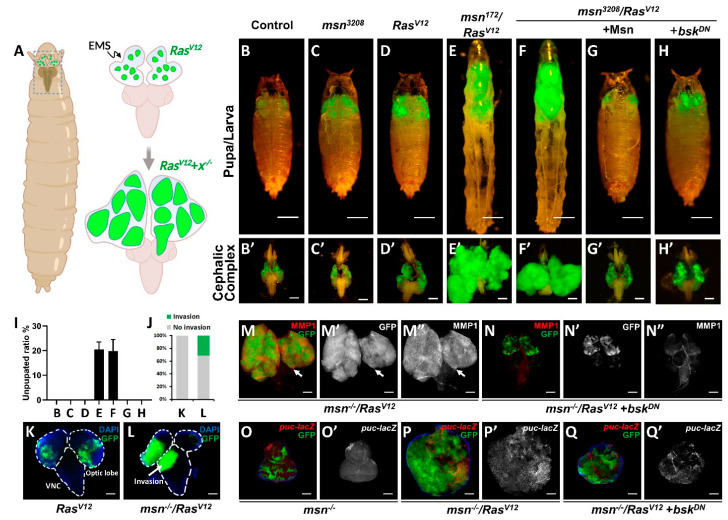
Loss of *Misshapen* (*msn*) synergizes with oncogenic *Ras* (RasV12) to induce tumorigenesis and invasion. (**A**) Strategy of an ethyl methanesulphonate (EMS)-induced forward genetic screen in developing *Drosophila* eye to identify novel *Ras^V12^* collaborating tumor suppressors. (**B**–**H**) Fluorescence micrographs of GFP-labeled pupa/larva are shown in the top panels, cephalic complexes dissected from third-instar larvae are shown in the bottom. Compared with control (**B**,**B**’), loss of *msn* alone has no obvious overgrowth (**C**,**C**’), ectopic expression of *Ras^V12^* alone only induces mild tumor growth (**D**,**D**’). *msn*^−/−^/*Ras^V12^* tumors display significant overgrowth (**E**,**F** and **E**’,**F**’), which can be rescued by co-expression of Msn (**G**,**G**’) or co-expression of a dominant negative form of *basket* (**H**,**H**’). (**I**) Quantification of unpupated ratio for indicated genotypes in **B**–**H**. (**J**–**L**) Fluorescence micrographs of GFP-labeled Optic lope and ventral nerve cord (VNC) dissected from third-instar larvae are shown, arrow indicates invasion sites (**K**,**L**). (**J**) Quantification of invasion ratio in (**K**) and (**L**). (**M**–**Q**) Loss of *msn* collaborates with *Ras^V12^* to induce tumorigenesis through activating c-Jun N-terminal kinase (JNK) signaling pathway. Expression of *basket* (*bsk^DN^* ) suppressed *msn*^−/−^*/Ras^V12^*-induced MMP1 expression, tumor invasion (**M**–**N**”), and upregulation of *puc* transcription (**O**–**Q**’); note that loss of msn alone does not significantly induce MMP1 activation (**O**,**O**’). Scale bars represent 500 μm (**B**–**H**), 200 μm (**B**’–**H**’, **M**–**M**”, and **N**–**N**”), or 100 μm (**K**–**L**, **O**–**Q**, and **O**’–**Q**’).

In order to identify novel tumor suppressors that can cooperate with *Ras^V12^* to promote tumor growth and invasion, we performed a large-scale EMS-induced genetic screen on *Drosophila* eye-antennal imaginal discs utilizing the *ey*-FLP-based mosaic analysis with repressible cell marker (MARCM) technique [16,22] (Figure 1A). Here, we identified a recessive-lethal allele (#3208) that exhibited invasive tumor overgrowth phenotype (Figure 1B,D,F). Subsequent deficiency mapping and complementation test revealed that #3208 allele disrupted the *misshapen* (CG16973) gene (labeled as *msn^3208^* thereafter). Consistent with this, we found that null allele of *msn* (*msn^172^*) [23] also synergized with *Ras^V12^* and exhibited similar degree of tumor overgrowth (Figure 1E). More importantly, the overgrowth phenotype and reduced pupation caused by *msn^3208^*/*Ras^V12^* were both completely rescued by co-expression of wild type Msn (Figure 1G,I and Supplementary Materials Figure S1L). We further validated the phenotype of *msn* mutant clones, and found that compared with wild type clones, loss of *msn* alone did not show significant growth advantage or changes in apoptosis, as indicated by PH3 and Caspase 3 staining, respectively (Figure 1B,C, Figure S1A,B,E,F,I–K,M). In accordance with previous reports [16,22], *Ras^V12^* expression alone only showed mild tumor overgrowth and did not invade into the ventral nerve cord (VNC) (Figure 1D,K), a commonly used tissue for tumor invasion observation in *Drosophila* [12,14,16,22], whereas larvae bearing *msn^3208^*/*Ras^V12^* tumors exhibited dramatic tumor overgrowth (Figure 1F,F’), autonomous cell proliferation (Figure S1G,H,M), reduced pupation ratio (Figure 1I), and increased VNC invasion behavior (Figure 1J,L). In contrast, we did not observe a significant change in apoptosis (Figure S1C,D). Consistent with increased tumor invasion, we also observed intensive MMP1 activation, a protein essential for basement membrane degradation and epithelial–mesenchymal transition (EMT) [24,25], in both primary tumor and invasive leading edges (Figure 1M). Taken together, these findings suggest that *msn* is a tumor suppressor that cooperatively induces tumor growth and invasion with *RAS^V12^*. 

### 2.2. msn^−/−^ Collaborates with Ras^V12^ to Activate JNK Signaling

Given that MMP1 also acts as a transcriptional target of JNK signaling pathway [24,25], the increase of MMP1 in *msn^3208^*/*Ras^V12^* tumors implies that JNK activation might be essential for tumorigenesis. Inconsequently, previous studies indicate that Msn acts as an MKKKK to activate the c-Jun N-terminal kinase (JNK) signaling pathway [23,26,27]. Our research also shows that ectopic Msn overexpression indeed induced mild JNK activation (Figure 3A,B), whereas loss of *msn* alone had no significant change on *puc* transcription (Figure 1O), another canonical JNK pathway target [25]. Intriguingly, we found loss of *msn* synergized with *Ras^V12^* to induce intensive MMP1 and *puc* activation (Figure 1M,P), both of which were significantly suppressed by expression of a dominant-negative form of *Drosophila* JNK homologue *basket* (*bsk^DN^*) (Figure 1H,N,Q and Figure S1L). It is also worth noting that although inhibition of JNK activity completely blocked *msn^−/−^*/*Ras^V12^*-induced *puc* upregulation in a cell-autonomous manner, we detected strong non-autonomous JNK activation in the surrounding region (Figure 1Q), which is probably caused by JNK propagation [28]. Together, these data indicate that loss of *msn* synergizes with *Ras^V12^* to promote tumor overgrowth via activating JNK signaling in vivo.

### 2.3. Loss of msn Synergizes with Ras^V12^ to Inactivate Hippo Pathway

**Figure 2:**
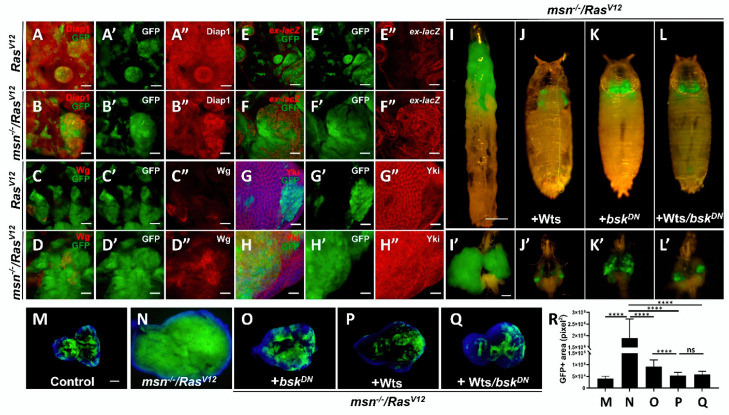
Loss of *msn* collaborates with *Ras^V12^* to inactivate Hippo pathway. (**A**–**H**) Fluorescence micrographs of GFP-labeled clones of eye discs are shown. Compared with *Ras^V12^* clones, *msn*^−/−^/*Ras^V12^* tumors show dramatic upregulation of Diap1 (**B**–**B**”), *ex-lacZ* (**D**–**D**”), Wg (**F**–**F**”), and mammalian homologue YAP/TAZ (Yki) localization (**H**–**H**”). (**I**–**L**) Fluorescence micrographs of GFP-labeled pupa/larva and cephalic complexes are shown. *msn^−/^*^−^/*Ras^V12^*-induced tumor growth phenotype is completely suppressed by mammalian homologue Lats 1/2 (Wts) overexpression with or without JNK blocking. (**M**–**R**) Ectopic Wts overexpression causes stronger suppression of *msn^−/^*^−^/*Ras^V12^* tumor growth phenotype than that of JNK inhibition alone (**M**–**Q**). Quantification of GFP-positive area of indicated genotypes (**R**). **** *p* < 0.0001(mean + S.D.). Scale bars represent 50 μm (**A**–**F**), 20 μm (**G**,**H**), 500 μm (**I**–**L**), 200 μm (**I**’–**L**’), 100 μm (**M**–**Q**).

Recent studies in *Drosophila* gut have uncovered a genetic link between the STE20 kinase Msn and the Hippo pathway, indicating that Msn may act in parallel with mammalian homologue MST 1/2 (Hpo), the key component of Hippo pathway, to regulate mammalian homologue Lats 1/2 (Wts) and mammalian homologue YAP/TAZ (Yki) activity [29,30,31,32,33,34]. Given that Hippo pathway plays a key role in regulating tumorigenesis [18,20,21], we hypothesized that the synergistic effect in *msn^−/−^*/*Ras^V12^* tumors might be caused by inactivation of Hippo signaling. We found that loss of *msn* alone in the developing eye disc showed neither changes in the endogenous expression of Hippo pathway target gene including Diap1 (Figure S2A,B), *ex* (Figure S2C,D), and Wg (Figure S2E,F), nor Yki localization (Figure S2G–H), while overexpression of *Ras^V12^* alone mildly upregulated Hippo target genes (Figure 2A,C,E) and Yki nucleus localization (Figure 2G); it is also worth noting that overall Yki levels were decreased in *Ras^V12^* clones (Figure 2G and Figure S2I). In *msn^−/−^*/*Ras^V12^* tumors, we observed intensive upregulation of Diap1, Wg, and *ex* (Figure 2B,D,F), as well as strong Yki nucleus localization (Figure 2H). Interestingly, we found that Wts overexpression not only dramatically blocked *msn^−/−^*/*Ras^V12^*-induced tumor growth (Figure 2I–J), but also further enhanced the tumor suppression phenotype caused by JNK inhibition (Figure 2K,L,P–R). Together, these data indicate that *msn^−/−^*/*Ras^V12^* drives tumor growth via Hippo signaling inactivation in vivo.

### 2.4. Msn Positively Regulates Hippo Signaling in Tumorigenesis

**Figure 3:**
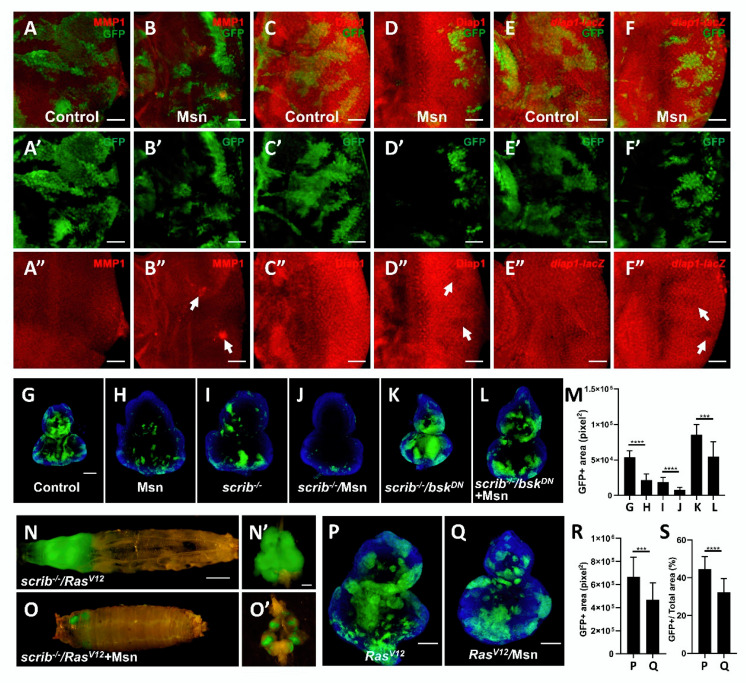
Msn positively regulates Hippo signaling. (**A**–**F, A’**–**F’** and **A’’**–**F’’**) Fluorescence micrographs of eye discs are shown. Compared with wild type (**A**–**A’’**, **C**–**C’’** and **E**–**E’’**), Msn overexpression induces mild JNK activation (**B**–**B’’**) and Hippo target gene downregulation (**D**–**D’’** and **F**–**F’’**). (**G**–**L**) Fluorescence micrographs of GFP-labeled clones of eye discs are shown. Msn overexpression decreases wild type clone size (**G** and **H**), *scribble* (*scrib)^−/−^* clone size (**I** and **J**), and significantly impedes *scrib^−/−^*/*bsk^DN^*-induced clone overgrowth (**K** and **L**). (**M**) Quantification of GFP-positive area of indicated genotypes. (**N**,**O**) Msn overexpression suppresses *Ras^V12^*/*scrib^−/−^* tumor growth. (**P**–**S**) Msn overexpression suppresses *Ras^V12^* absolute and relative clone size (**P** and **Q**). Quantification of GFP-positive absolute (**R**) and relative area (**S**) of indicated genotypes. *** *p* < 0.001 (mean + S.D.); **** *p* < 0.0001 (mean + S.D.). Scale bars represent 50 μm (**A**–**F**), 100 μm (**G**–**L** and **P**,**Q**), 500 μm (**N**,**O**), 200 μm (**N**’,**O**’).

Previous studies indicated that Msn can functionally substitute for Hippo to restrict Yki activity in intestine [29,30,31]. Consistent with this notion that Msn is a positive regulator of Hippo pathway, we found Msn overexpression alone caused clone undergrowth (Figure 3G,H,M) and significantly reduced endogenous expression of Yki reporter Diap1 (Figure 3C,D and Figure S3G,H) and *diap1-lacZ* (Figure 3E,F and Figure S3I,J) in *Drosophila* imaginal discs, whereas it had no obvious changes on apoptosis and mitosis (Figure S3A–D). In *Drosophila*, cell polarity gene *scribble* (*scrib*) acts as a neoplastic tumor suppressor and would be eliminated by Hippo-mediated cell competition [35,36]. We found that Msn overexpression caused a further reduction of *scrib^−/−^* clone sizes (Figure 3I,J,M). It has been proven that the elimination of *scrib^−/−^* clone depends on JNK-activation-induced Yki inhibition, and given that Msn overexpression can activate both JNK and Hippo signaling (Figure 3A,B), we blocked JNK activity and simultaneously overexpressed Msn in *scrib^−/−^* clones. We found that the overgrowth phenotype of *scrib^−/−^/bsk^DN^* clone (Figure 3K) was significantly reduced by co-expression of Msn (Figure 3L,M), indicating that Msn promotes *scrib^−/−^* elimination in a JNK-independent manner. In accordance with this, we also found that Msn overexpression significantly suppressed *Ras^V12^/scrib^−/−^*-induced tumor overgrowth (Figure 3N,O and Figure S3E,F), which has been proven to be mediated by Hippo signaling [37,38,39]. Moreover, we found that Msn overexpression reduced both the relative and absolute clone sizes of *Ras^V12^* (Figure 3P–S). Together, these results indicate that ectopic Msn overexpression activates Hippo signaling to suppress tumorigenesis.

### 2.5. Msn Acts Downstream of Ft in Modulating Hippo Signaling

**Figure 4:**
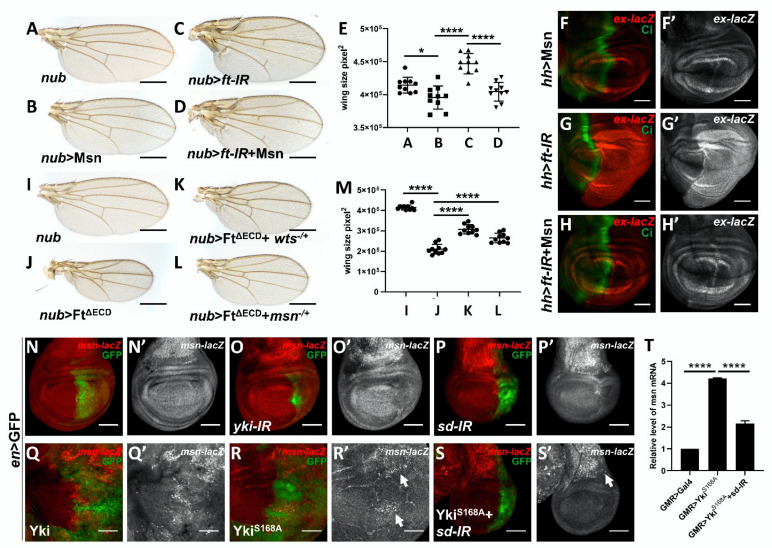
Msn acts downstream of protocadherin Fat (Ft) in modulating Hippo signaling. (**A**–**E**) Light micrographs of *Drosophila* adult wings. Compared with control (**A**), Msn overexpression significantly impedes *ft* knockdown-induced overgrowth phenotype (**C** and **D**); note that overexpression of Msn alone mildly reduces wing size (**B**). (**E**) Quantification of wing size in (**A**–**D**). (**F**–**H** and **F’**–**H’**) Fluorescence micrographs of wing discs are shown. Msn overexpression does not significantly affect *ex* transcription under *hh*-Gal4 (**F** and **F’**), but significantly suppresses *ft* knockdown-induced *ex* upregulation (**G**–**G’** and **H**–**H’**). (**I**–**L**) Light micrographs of *Drosophila* adult wings are shown. Removing one copy of *wts* (**K**) or *msn^−/+^* (**L**) significantly impedes *nub* > Ft*^ΔECD^*-induced wing undergrowth phenotype (**J**). (**M**) Quantification of wing size in (**I**–**L**). (**N**–**S**) Fluorescence micrographs of wing discs are shown. Compared with control (**N** and **N**’), knockdown *yki* or *sd* reduce endogenous *msn* transcription (**O**,**P** and **O**’,**P**’); Yki or Yki^S168A^ overexpression upregulates *msn* transcription (**Q**–**R**’), which is suppressed by knocking down *sd* (**S**). (**T**) Quantification of relative level of *msn* mRNA of indicated genotypes. * p < 0.05 (mean + S.D.); **** *p* < 0.0001 (mean + S.D.). Scale bars represent 200 μm (**A**–**D** and **I**–**L**), 100 μm (**F**–**H**, **N**–**S**, and **N**’–**S**’).

Although we and others proved that Msn positively activates Hippo signaling in various fly organs, the upstream regulator, especially the membrane anchor of Msn, remains unclear [31]. Fat signaling has previously been shown to be involved in both the establishment of planar cell polarity and growth control, Ft is a transmembrane protein that negatively regulates organ size and functions as a well-established, essential regulator of Hippo signaling in both eye and wing disc [19,40,41,42,43]. Because the quantification of wing size is relatively easy and standard deviation is lower, we dissected the genetic interactions between Msn and Ft in developing wing, with specific focus on the overall size of the adult wing [43]. As previously shown, knockdown of *ft* expression in the wing pouch region using *nub*-Gal4 results in a significant increase in wing size [44] (Figure 4A,C,E), while overexpression of Msn alone results in a significant decrease in wing size (Figure 4B,E), consistent with its role as a positive regulator of Hippo signaling. When we knocked down *ft* and simultaneously overexpressed Msn, the wing size phenocopies that of Msn overexpression alone (Figure 4D–E). Similarly, we found that *ft* knockdown-induced *ex* upregulation was also suppressed when Msn was co-expressed (Figure 4F–H and Figure S4B). To further dissect the physiological function of *msn* in regulating Ft-mediated growth, we removed one copy of *msn*, and found that ectopic expression of truncated form of Ft that removed most of the extracellular domains (Ft^ΔECD^) caused small wing phenotype (Figure 4J); this reduction in wing size was suppressed by heterozygosity of the *msn* allele (Figure 4L), phenocopying that of *wts* deletion (Figure 4K). Taken together, these genetic data indicate that Msn acts downstream of Ft in modulating Hippo signaling.

Interestingly, a protein–protein interaction screen in fly uncovered a potential physical link between Ft and Msn [45]; therefore, we further examined the physical interaction between Ft and Msn in S2 cells. Unfortunately, our co-immunoprecipitation experiment did not detect a physical binding between Ft^ΔECD^ and Msn, suggesting that Ft might act through other unknown protein(s) to regulate Msn-mediated Hippo activation (Figure S4C,D).

### 2.6. Msn Acts as a Hippo Target Gene in a Negative Feedback Manner

Given that human ortholog of Msn (MAP4K4) is overexpressed in various human cancers [46,47,48,49,50] and a number of Hippo pathway components regulate Hippo signaling in a negative feedback manner, including *kibra*, *ex*, *wts* [18,20,21,51], we utilized the *Drosophila* wing imaginal discs to explore whether Msn also functions as a Hippo pathway target gene. Compared with control, knockdown Hippo signaling effector Yki or the transcription factor Scalloped (Sd) under *en*-Gal4 significantly reduced the Msn transcription level, as demonstrated by a lacZ enhancer trap insertion within the *msn* locus (Figure 4N–P). Conversely, Yki and active form of Yki (Yki^S168A^) overexpression significantly upregulated *msn* transcription level (Figure 4Q,R) and co-expression of an *sd RNAi* significantly impeded Yki^S168A^-induced *msn-lacZ* upregulation (Figure 4S). Interestingly, we also found that Msn overexpression suppressed transcription of itself (Figure S4A). To further assess the role of Yki and Sd in regulating *msn* expression, we performed qRT-PCR in vivo using adult fly heads, which are relatively easy to collect and extract mRNA from for qPCR analysis. Consistent with wing disc results, Yki overexpression in the developing eye disc upregulated *msn* transcription, which was significantly impaired by knocking down *sd* (Figure 4T). Taken together, these data indicate that Msn also acts as a Yki/Sd target to form a negative feedback loop.

## 3. Discussion

*Drosophila* has been widely used as a cancer model for the past few decades to dissect various human cancer biology questions, including cell–cell communication, tumor heterogeneity, clonal evolution, cancer cachexia, and antitumor drug resistance [5,6,7]. The powerful genetic tools established in *Drosophila*, especially the mosaic analysis with a repressible cell marker (MARCM) system [52,53], make it possible to perform large-scale genetic screens aimed at identifying novel tumor suppressor genes in vivo [12,16,22]. In this study, we conducted an EMS-induced unbiased modified genetic screen and identified *misshapen* (CG16973) as a tumor suppressor that synergizes with *Ras^V12^* to drive tumor overgrowth by inactivating Hippo signaling in *Drosophila* (Figure S5). 

Misshapen (Msn) is a member of the STE20 kinase family; it was initially identified as a MAP kinase kinase kinase kinase (MKKKK) that activates the c-Jun N-terminal kinase (JNK) pathway [23,26,27]. Msn also regulates diverse physiological functions including dorsal closure, photoreceptor axon targeting, germline ring canal stabilization, and intestine homeostasis [23,29,31,54,55]. Moreover, recent studies have shown that *Drosophila* Msn and its mammalian homologue MAP4K4/6/7 act in parallel and partially redundantly with canonical Hippo signaling component mammalian homologue MST 1/2 (Hpo) in regulating mammalian homologue Lats 1/2 (Wts) and mammalian homologue YAP/TAZ (Yki) [29,30,31,32,33,34,56,57]. Given that Hippo signaling pathway is an evolutionarily conserved pathway that regulates various essential physiological processes and its disruption contributes to a number of human diseases including cancer [18,20,21,58], it is possible that Msn and its mammalian homologue MAP4K4/6/7 may also function as a tumor suppressor in a context-dependent manner.

*RAS* family genes are considered to be one of the most highly mutated genes in various cancers [1,2,3,4]; however, treatment targeting *RAS*-related tumors remains unsatisfactory, and the in vivo molecular mechanisms of *RAS*-related tumorigenesis are still not completely understood. Here, we identified *msn* as a tumor suppressor that cooperates with oncogenic *Ras^V12^* to promote tumor overgrowth and invasion by simultaneously activating JNK pathway and inactivating Hippo pathway. We found that Msn overexpression dramatically suppressed *scrib^1^*/*Ras^V12^*-induced tumor overgrowth and invasion, a well-established *Drosophila* cancer model. Additionally, we revealed that *msn* acts downstream of Ft and regulates Hippo pathway in a negative feedback manner. It is worth noting that we could not exclude the possibility that *msn^−/−^/Ras^V12^* tumors could also regulate other growth regulating pathways, including JAK-STAT signaling, which requires further investigation (Figure S5).

Given the high conservation of signaling pathways and cancer-related genes between *Drosophila* and human, we assume that similar mechanisms could be involved in human cancer progression. Our study here identified Msn as a tumor suppressor and further investigation in mammal and human may provide potential therapeutic targets for cancer treatment, especially for Hippo-related tumors.

## 4. Experimental Procedures

### 4.1. Drosophila Stocks and Genetics

All crosses were raised on standard *Drosophila* media at 25 °C unless otherwise indicated. Fluorescently labeled clones were produced in the eye discs as previously described [12,16,22] using the following strains: *ey*-Flp1; *Act > y+ >* Gal4, *UAS*-GFP; *tub*-Gal80, FRT79E (79E tester); *ey*-Flp1; *Act*> *y+* > Gal4, *UAS*-GFP; *tub*-Gal80, FRT80B (80B tester); *ey*-Flp5, *Act* > *y+* > Gal4, *UAS*-GFP; FRT82B, *tub*-Gal80 (82B tester). Additional strains, including *GMR*-Gal4, *en*-Gal4, *hh*-Gal4, *nub*-Gal4, *UAS*-GFP, *UAS-ft-IR*, *wts^X1^* (#44251), *msn^172^* (#5947), *msn*-*LacZ* (#11707), and *UAS*-Msn (#5946), were obtained from Bloomington *Drosophila* Stock Center; *puc^E69^*, *UAS*-*Ras^V12^* [15], *UAS*-*bsk^DN^* [17], and *scrib^1^* [59] were previously described. *UAS*-Wts, *UAS*-Yki, *UAS*-Yki^S168A^, *UAS-yki-IR*, and *UAS-sd-IR* were previously described [60]. *UAS*-Ft^ΔECD^ is a kind gift from Kenneth Irvine.

### 4.2. EMS Mutagenesis and Genetic Screen

We focused on *Drosophila* chromosome 3 L in this screen. Male flies carrying FRT79E (Sp/CyO-GFP; FRT79E) were starved for 8 h and subsequently fed with 25 mM EMS solution overnight at room temperature. The mutagenized males were then mated to females of the genotype *UAS*-*Ras^V12^*; sb/TM6B. Single F1 males of the genotype *UAS*-*Ras^V12^*/CyO-GFP; ∗FRT79E/TM6B were crossed to Sp/CyO; sb/TM6B first and then crossed with the 79E tester line for validation of GFP-labeled tumor overgrowth phenotype under fluorescent microscope.

### 4.3. Immunostaining

Third-instar larvae eye-antennal discs were dissected in 1 × PBS, fixed in freshly made 4% paraformaldehyde, and stained as described previously [61] using the following primary antibodies: mouse anti-MMP1 (1:200, Developmental Studies Hybridoma Bank, DSHB, Iowa City, IA, USA), mouse anti-β-Gal (1:1000, Promoga, Madison, WI, USA), rabbit anti-phospho-histone 3 (PH3) (1:200, Cell Signaling Technology, CST, Danvers, MA, USA), rabbit anti-active caspase-3 (1:400, Cell Signaling Technology, CST, Danvers, MA, USA), mouse anti-Diap1 (1:200, a gift from Bruce Hay, California Institute of Technology, Pasadena, CA, USA), rabbit anti-Yki (1:500, gift from Duojia Pan, University of Texas Southwestern Medical Center, Dallas, TX, USA), and mouse anti-Wg (1:200, Developmental Studies Hybridoma Bank, DSHB, Iowa City, IA, USA). Secondary antibodies were anti-rabbit-Cy3 (1:400, Thermo Fisher Scientific, Waltham, MA, USA) and anti-mouse-Cy3 (1:400, Thermo Fisher Scientific, Waltham, MA, USA). 

### 4.4. Cell Culture

S2 cells were maintained in Schneider’s Drosophila Medium (Gibco #21720024) supplemented with 10% fetal bovine serum (Cellmax #SA112) and 1% penicillin–streptomycin (Gibco #15140122) at 28 °C.

### 4.5. Immunoprecipitation and Western Blotting

Transfection, Co-IP, and Western blot analysis were performed as previously described with some modification [62]. paw-Gal4, UAS-attB-msn-HA, and UAS-attB-Fat^ΔECD^-FLAG plasmids were co-transfected to S2 cells, performed using Effectene transfection reagent (Qiagen #301427) following the manufacturer’s instructions and harvested 72 h after transfection. Harvested S2 cells were lysed in 500 µL RIPA lysis buffer (50 mM Tris pH 7.5, 150 mM NaCl, 1 mM EDTA, 1% NP40, 5% glycerol, protease inhibitor cocktail, DTT). After the samples were rotated for 1 h at 4 °C, lysates were centrifuged at 13000 rpm for 13 min at 4 °C. The lysate supernatant was incubated at 4 °C with 20 µL cleaned anti-FLAG M2 Affinity Gel (Sigma #A2220) for 3 h. The beads were washed with PBST four times, then mixed with SDS-PAGE Loading Buffer (reducing, 5×) (CWBIO #CW0027S) and heated to 95 °C for 7 min. After separation of proteins by SDS-PAGE, proteins were transferred to PVDF membrane (Merck Millipore #IPVH00010) which was blocked with 5% milk–PBST for 1 h, incubated in primary antibody overnight at 4 °C, washed with PBST, incubated with secondary antibody at room temperature for 1 h, and washed with PBST four times. Protein was detected using a Viber Fusion FX6 Spectra imaging system (Vilber, Collégien, France). The following antibodies for Western blotting were used: primary antibodies, rabbit-anti-FLAG (1:10,000) (EASYBIO #BE2005); rabbit-anti–HA (1:9000) (EASYBIO #BE2006); and secondary antibody anti-rabbit-HRP (1:8000) (Promega #W4011).

### 4.6. Quantitative Real-Time PCR

*Drosophila* adult head of indicated genotypes was removed and total RNA was then extracted using TRIzol (Ambion). Total RNA was reverse-transcribed into cDNA with the HiScript II 1st Strand cDNA Synthesis Kit (Vazyme); and quantitative PCR was performed with KAPA SYBR^®^ FAST (KAPA BIOSYSTEMS) and quantified by the QuantStudio™ 5 Real-Time PCR System (ThermoFisher). RP49 was used as an internal control. The following primer sequences were used for real-time PCR. *msn*: F: 5′-TCCCTTGGACAGCAGCGATT-3′, R: 5′-AGTTCCATCGTTCCTAGCC-3′; *rp49*: F: 5′-TCCTACCAGCTTCAAGATGACC-3′, R: 5′-CACGTTGTGCACCAGGAACT-3′.

### 4.7. Statistical Analysis

Clone and wing size were measured with ImageJ and Photoshop, respectively. Quantification of the data was presented in bar graphs created with GraphPad Prism 8. Data represent mean values + SD. We used Mann–Whitney U test for multiple comparisons to calculate statistical significance (* *p* < 0.05; ** *p* < 0.01; *** *p* < 0.001; **** *p* < 0.0001).

## Supplementary Materials

The following are available online at https://www.mdpi.com/article/10.3390/cells10040894/s1, Figure S1: Loss of *msn* collaborates with *Ras^V12^* to induce tumorigenesis. Figure S2: Loss of msn alone does not affect Hippo signaling activation. Figure S3: Msn overexpression suppresses tumorigenesis. Figure S4: Msn regulates Hippo signaling in a feedback manner. Figure S5: A schematic model depicting the role of Msn in regulating Hippo signaling and tumorigenesis.

## References

[1] Ryan M.B., Der C.J., Wang-Gillam A., Cox A.D. (2015). Targeting RAS-mutant cancers: Is ERK the key?. Trends Cancer.

[2] Stephen A.G., Esposito D., Bagni R.K., McCormick F. (2014). Dragging ras back in the ring. Cancer Cell.

[3] Cox A.D., Fesik S.W., Kimmelman A.C., Luo J., Der C.J. (2014). Drugging the undruggable RAS: Mission possible?. Nat. Rev. Drug Discov..

[4] Samatar A.A., Poulikakos P.I. (2014). Targeting RAS-ERK signalling in cancer: Promises and challenges. Nat. Rev. Drug Discov..

[5] Saavedra P., Perrimon N. (2019). Drosophila as a Model for Tumor-Induced Organ Wasting. Adv. Exp. Med. Biol..

[6] Enomoto M., Siow C., Igaki T. (2018). Drosophila As a Cancer Model. Adv. Exp. Med. Biol..

[7] Mirzoyan Z., Sollazzo M., Allocca M., Valenza A.M., Grifoni D., Bellosta P. (2019). Drosophila melanogaster: A Model Organism to Study Cancer. Front. Genet..

[8] Richardson H.E., Cordero J.B., Grifoni D. (2020). Basic and Translational Models of Cooperative Oncogenesis. Int. J. Mol. Sci..

[9] Pastor-Pareja J.C., Xu T. (2013). Dissecting social cell biology and tumors using Drosophila genetics. Annu. Rev. Genet..

[10] Gonzalez C. (2013). Drosophila melanogaster: A model and a tool to investigate malignancy and identify new therapeutics. Nat. Rev. Cancer.

[11] Figueroa-Clarevega A., Bilder D. (2015). Malignant Drosophila tumors interrupt insulin signaling to induce cachexia-like wasting. Dev. Cell.

[12] Pagliarini R.A., Xu T. (2003). A genetic screen in Drosophila for metastatic behavior. Science.

[13] Brumby A.M., Richardson H.E. (2003). scribble mutants cooperate with oncogenic Ras or Notch to cause neoplastic overgrowth in Drosophila. EMBO J..

[14] Igaki T., Pagliarini R.A., Xu T. (2006). Loss of cell polarity drives tumor growth and invasion through JNK activation in Drosophila. Curr. Biol..

[15] Ma X., Li W., Yu H., Yang Y., Li M., Xue L., Xu T. (2014). Bendless modulates JNK-mediated cell death and migration in Drosophila. Cell Death Differ..

[16] Ma X., Lu J.Y., Dong Y., Li D., Malagon J.N., Xu T. (2017). PP6 Disruption Synergizes with Oncogenic Ras to Promote JNK-Dependent Tumor Growth and Invasion. Cell Rep..

[17] Ma X., Xu W., Zhang D., Yang Y., Li W., Xue L. (2015). Wallenda regulates JNK-mediated cell death in Drosophila. Cell Death Dis..

[18] Pan D. (2010). The hippo signaling pathway in development and cancer. Dev. Cell.

[19] Staley B.K., Irvine K.D. (2012). Hippo signaling in Drosophila: Recent advances and insights. Dev. Dyn..

[20] Zeng R., Dong J. (2021). The Hippo Signaling Pathway in Drug Resistance in Cancer. Cancers.

[21] Zheng Y., Pan D. (2019). The Hippo Signaling Pathway in Development and Disease. Dev. Cell.

[22] Ma X., Lu J.Y., Moraru A., Teleman A.A., Fang J., Qiu Y., Liu P., Xu T. (2020). A novel regulator of ER Ca(2+) drives Hippo-mediated tumorigenesis. Oncogene.

[23] Su Y.C., Treisman J.E., Skolnik E.Y. (1998). The Drosophila Ste20-related kinase misshapen is required for embryonic dorsal closure and acts through a JNK MAPK module on an evolutionarily conserved signaling pathway. Genes Dev..

[24] Srivastava A., Pastor-Pareja J.C., Igaki T., Pagliarini R., Xu T. (2007). Basement membrane remodeling is essential for Drosophila disc eversion and tumor invasion. Proc. Natl. Acad. Sci. USA.

[25] Uhlirova M., Bohmann D. (2006). JNK- and Fos-regulated Mmp1 expression cooperates with Ras to induce invasive tumors in Drosophila. EMBO J..

[26] Liu H., Su Y.C., Becker E., Treisman J., Skolnik E.Y. (1999). A Drosophila TNF-receptor-associated factor (TRAF) binds the ste20 kinase Misshapen and activates Jun kinase. Curr. Biol..

[27] Paricio N., Feiguin F., Boutros M., Eaton S., Mlodzik M. (1999). The Drosophila STE20-like kinase misshapen is required downstream of the Frizzled receptor in planar polarity signaling. EMBO J..

[28] Wu M., Pastor-Pareja J.C., Xu T. (2010). Interaction between Ras(V12) and scribbled clones induces tumour growth and invasion. Nature.

[29] Li Q., Li S., Mana-Capelli S., Roth Flach R.J., Danai L.V., Amcheslavsky A., Nie Y., Kaneko S., Yao X., Chen X. (2014). The conserved misshapen-warts-Yorkie pathway acts in enteroblasts to regulate intestinal stem cells in Drosophila. Dev. Cell.

[30] Li Q., Nirala N.K., Chen H.J., Nie Y., Wang W., Zhang B., Czech M.P., Wang Q., Xu L., Mao J. (2019). The Misshapen subfamily of Ste20 kinases regulate proliferation in the aging mammalian intestinal epithelium. J. Cell. Physiol..

[31] Li Q., Nirala N.K., Nie Y., Chen H.J., Ostroff G., Mao J., Wang Q., Xu L., Ip Y.T. (2018). Ingestion of Food Particles Regulates the Mechanosensing Misshapen-Yorkie Pathway in Drosophila Intestinal Growth. Dev. Cell.

[32] Li S., Cho Y.S., Yue T., Ip Y.T., Jiang J. (2015). Overlapping functions of the MAP4K family kinases Hppy and Msn in Hippo signaling. Cell Discov..

[33] Meng Z., Moroishi T., Mottier-Pavie V., Plouffe S.W., Hansen C.G., Hong A.W., Park H.W., Mo J.S., Lu W., Lu S. (2015). MAP4K family kinases act in parallel to MST1/2 to activate LATS1/2 in the Hippo pathway. Nat. Commun..

[34] Meng Z., Qiu Y., Lin K.C., Kumar A., Placone J.K., Fang C., Wang K.C., Lu S., Pan M., Hong A.W. (2018). RAP2 mediates mechanoresponses of the Hippo pathway. Nature.

[35] Chen C.L., Schroeder M.C., Kango-Singh M., Tao C., Halder G. (2012). Tumor suppression by cell competition through regulation of the Hippo pathway. Proc. Natl. Acad. Sci. USA.

[36] Nagata R., Igaki T. (2018). Cell competition: Emerging mechanisms to eliminate neighbors. Dev. Growth Differ..

[37] Doggett K., Grusche F.A., Richardson H.E., Brumby A.M. (2011). Loss of the Drosophila cell polarity regulator Scribbled promotes epithelial tissue overgrowth and cooperation with oncogenic Ras-Raf through impaired Hippo pathway signaling. BMC Dev. Biol..

[38] Enomoto M., Igaki T. (2011). Deciphering tumor-suppressor signaling in flies: Genetic link between Scribble/Dlg/Lgl and the Hippo pathways. J. Genet. Genomics.

[39] Fahey-Lozano N., La Marca J.E., Portela M., Richardson H.E. (2019). Drosophila Models of Cell Polarity and Cell Competition in Tumourigenesis. Adv. Exp. Med. Biol..

[40] Thomas C., Strutt D. (2012). The roles of the cadherins Fat and Dachsous in planar polarity specification in Drosophila. Dev. Dyn..

[41] Montes A.J., Morata G. (2017). Homeostatic response to blocking cell division in Drosophila imaginal discs: Role of the Fat/Dachsous (Ft/Ds) pathway. Dev. Biol..

[42] Cho E., Feng Y., Rauskolb C., Maitra S., Fehon R., Irvine K.D. (2006). Delineation of a Fat tumor suppressor pathway. Nat. Genet..

[43] Willecke M., Hamaratoglu F., Kango-Singh M., Udan R., Chen C.L., Tao C., Zhang X., Halder G. (2006). The fat cadherin acts through the hippo tumor-suppressor pathway to regulate tissue size. Curr. Biol..

[44] Verghese S., Waghmare I., Kwon H., Hanes K., Kango-Singh M. (2012). Scribble acts in the Drosophila fat-hippo pathway to regulate warts activity. PLoS ONE.

[45] Kwon Y., Vinayagam A., Sun X., Dephoure N., Gygi S.P., Hong P., Perrimon N. (2013). The Hippo signaling pathway interactome. Science.

[46] Hao J.M., Chen J.Z., Sui H.M., Si-Ma X.Q., Li G.Q., Liu C., Li J.L., Ding Y.Q., Li J.M. (2010). A five-gene signature as a potential predictor of metastasis and survival in colorectal cancer. J. Pathol..

[47] Liang J.J., Wang H., Rashid A., Tan T.H., Hwang R.F., Hamilton S.R., Abbruzzese J.L., Evans D.B., Wang H. (2008). Expression of MAP4K4 is associated with worse prognosis in patients with stage II pancreatic ductal adenocarcinoma. Clin. Cancer Res..

[48] Qiu M.H., Qian Y.M., Zhao X.L., Wang S.M., Feng X.J., Chen X.F., Zhang S.H. (2012). Expression and prognostic significance of MAP4K4 in lung adenocarcinoma. Pathol. Res. Pract..

[49] Rizzardi A.E., Rosener N.K., Koopmeiners J.S., Isaksson Vogel R., Metzger G.J., Forster C.L., Marston L.O., Tiffany J.R., McCarthy J.B., Turley E.A. (2014). Evaluation of protein biomarkers of prostate cancer aggressiveness. BMC Cancer.

[50] Liu A.W., Cai J., Zhao X.L., Jiang T.H., He T.F., Fu H.Q., Zhu M.H., Zhang S.H. (2011). ShRNA-targeted MAP4K4 inhibits hepatocellular carcinoma growth. Clin. Cancer Res..

[51] Park G.S., Oh H., Kim M., Kim T., Johnson R.L., Irvine K.D., Lim D.S. (2016). An evolutionarily conserved negative feedback mechanism in the Hippo pathway reflects functional difference between LATS1 and LATS2. Oncotarget.

[52] Lee T., Luo L. (2001). Mosaic analysis with a repressible cell marker (MARCM) for Drosophila neural development. Trends Neurosci..

[53] Lee T., Luo L. (1999). Mosaic analysis with a repressible cell marker for studies of gene function in neuronal morphogenesis. Neuron.

[54] Kline A., Curry T., Lewellyn L. (2018). The Misshapen kinase regulates the size and stability of the germline ring canals in the Drosophila egg chamber. Dev. Biol..

[55] Su Y.C., Maurel-Zaffran C., Treisman J.E., Skolnik E.Y. (2000). The Ste20 kinase misshapen regulates both photoreceptor axon targeting and dorsal closure, acting downstream of distinct signals. Mol. Cell Biol..

[56] Zheng Y., Wang W., Liu B., Deng H., Uster E., Pan D. (2015). Identification of Happyhour/MAP4K as Alternative Hpo/Mst-like Kinases in the Hippo Kinase Cascade. Dev. Cell.

[57] Jiang J. (2018). Misshapen Connects Food, Mechanosensing, and Intestinal Growth. Dev. Cell.

[58] Dey A., Varelas X., Guan K.L. (2020). Targeting the Hippo pathway in cancer, fibrosis, wound healing and regenerative medicine. Nat. Rev. Drug Discov..

[59] Igaki T., Pastor-Pareja J.C., Aonuma H., Miura M., Xu T. (2009). Intrinsic tumor suppression and epithelial maintenance by endocytic activation of Eiger/TNF signaling in Drosophila. Dev. Cell.

[60] Ma X., Chen Y., Xu W., Wu N., Li M., Cao Y., Wu S., Li Q., Xue L. (2015). Impaired Hippo signaling promotes Rho1-JNK-dependent growth. Proc. Natl. Acad. Sci. USA.

[61] Ma X., Yang L., Yang Y., Li M., Li W., Xue L. (2013). dUev1a modulates TNF-JNK mediated tumor progression and cell death in Drosophila. Dev. Biol..

[62] Zhang Y., Wang X., Matakatsu H., Fehon R., Blair S.S. (2016). The novel SH3 domain protein Dlish/CG10933 mediates fat signaling in Drosophila by binding and regulating Dachs. Elife.

