# Supplementary material for: Misshapen Disruption Cooperates with RasV12 to Drive Tumorigenesis
Source: Cells. 2021 Apr 14;10(4):894. doi: 10.3390/cells10040894 (PMC8070713; doi:10.3390/cells10040894)
Supplement: Supplementary file 1 [file cells-10-00894-s001.pdf]

Misshapen Disruption Cooperates with *Ras*^{V12} to Drive Tumorigenesis

Du Kong, Jin-Yu Lu, Xiaoqin Li, Sihua Zhao, Wenyan Xu, Jinan Fang, Xing Wang, Xianjue Ma

Genotypes of all the figures

Supplemental figure1

Supplemental figure 2

Supplemental figure 3

Supplemental figure 4

Supplemental figure 5

Genotypes of all the figures

Figure 1

(B) *ey*-Flp1/+; *Act>y+*>Gal4, UAS-GFP/+; *tub*-Gal80, FRT79E/FRT79E; (C) *ey*-Flp1/+; *Act>y* +>Gal4, *UAS*-GFP/+; *tub*-Gal80, FRT79E/*msn*^{3208}, FRT79E; (D and K) *ey*-Flp1/+; *Act>y+*>Gal4, *UAS*-GFP/*UAS-Ras*^{V12}; *tub*-Gal80, FRT79E/ FRT79E; (E) *ey*-Flp1/+; *Act>y+*>Gal4, *UAS*-GFP/ *UAS-Ras*^{V12}; *tub*-Gal80, FRT80B/*msn*^{172}, FRT80B; (F, L-M) *ey*-Flp1/+; *Act>y+*>Gal4, *UAS*-GFP/ *UAS-Ras*^{V12}; *tub*-Gal80, FRT79E/*msn*^{3208}, FRT79E; (G) *ey*-Flp1/+; *Act>y+*>Gal4, *UAS*-GFP/*UAS-Ras*^{V12}; *tub*-Gal80, FRT79E/*msn*^{3208}, *UAS*-Msn, FRT79E; (H, N) *ey*-Flp1/*UAS-bsk*^{DN}; *Act>y+*> Gal4, *UAS*-GFP/*UAS-Ras*^{V12}; *tub*-Gal80, FRT79E/*msn*^{3208}, FRT79E; (O) *ey*-Flp1/+; *Act>y+*>Gal4, *UAS*-GFP/+; *tub*-Gal80, FRT79E, *puc*^{E69}/*msn*^{3208}, FRT79E; (P) *ey*-Flp1/+; *Act>y+*>Gal4, *UAS*-GFP/*UAS-Ras*^{V12}; *tub*-Gal80, FRT79E, *puc*^{E69}/*msn*^{3208}, FRT79E; (Q) *ey*-Flp1/UAS-*bsk*^{DN}; *Act>y+*> Gal4, *UAS*-GFP/*UAS-Ras*^{V12}; *tub*-Gal80, FRT79E, *puc*^{E69}/*msn*^{3208}, FRT79E.

Figure 2

(A, C and G) *ey*-Flp1/+; *Act>y+*>Gal4, *UAS*-GFP/*UAS-Ras*^{V12}; *tub*-Gal80, FRT79E/FRT79E; (B, D and H) *ey*-Flp1/+; *Act>y+*>Gal4, *UAS*-GFP/*UAS-Ras*^{V12}; *tub*-Gal80, FRT79E/*msn*^{3208}, FRT79E; (E) *ey*-Flp1/+; *Act>y+*>Gal4, *UAS*-GFP/*ex-lacZ*, *UAS-Ras*^{V12}; *tub*-Gal80, FRT79E/FRT79E; (F) *ey*-Flp1/+; *Act>y+*>Gal4, *UAS*-GFP/*ex-lacZ*, *UAS-Ras*^{V12}; *tub*-Gal80, FRT79E/*msn*^{3208}, FRT79E; (I and N) *ey*-Flp5, *Act>y+*> Gal4, *UAS*-GFP/*UAS-Ras*^{V12}; *tub*-Gal80, FRT79E/*msn*^{3208}, FRT79E; (J and P) *ey*-Flp5, *Act>y+*>Gal4, *UAS*-GFP/*UAS*-Wts, *UAS-Ras*^{V12}; *tub*-Gal80, FRT79E/*msn*^{3208}, FRT79E; (K and O) *UAS-bsk*^{DN}/+; *ey*-Flp5, *Act>y+*>Gal4, *UAS*-GFP/*UAS-Ras*^{V12}; *tub*-Gal80, FRT79E/*msn*^{3208}, FRT79E; (L and Q) *UAS-bsk*^{DN}/+; *ey*-Flp5, *Act>y+*> Gal4, *UAS*-GFP/*UAS*-Wts, *UAS-Ras*^{V12}; *tub*-Gal80, FRT79E/*msn*^{3208}, FRT79E; (M) *ey*-Flp5, *Act>y+*>Gal4, *UAS*-GFP/+; *tub*-Gal80, FRT79E/FRT79E.

Figure 3

(A, C and G) *ey*-Flp5, *Act>y+*>Gal4, *UAS*-GFP/+; FRT 82B, *tub*-Gal80/FRT82B; (B, D and H) *ey*-Flp5, *Act>y+*>Gal4, *UAS*-GFP/+; FRT 82B, *tub*-Gal80/FRT82B, *UAS*-Msn; (E) *ey*-Flp5, *Act> y+*> Gal4, *UAS*-GFP/+; *diap1-lacZ*, FRT 82B, *tub*-Gal80/FRT82B; (F) *ey*-Flp5, *Act>y+*>Gal4, *UAS*-GFP/+; *diap1-lacZ*, FRT 82B, *tub*-Gal80/FRT82B, *UAS*-Msn; (I) *ey*-Flp5, *Act>y+*>Gal4, *UAS*-GFP/+; FRT 82B, *tub*-Gal80/FRT82B, *scrib*^{1}; (J) *ey*-Flp5, *Act>y+*>Gal4, *UAS*-GFP/+; FRT 82B, *tub*-Gal80/FRT82B, *UAS*-Msn, *scrib*^{1}; (K) *UAS-bsk*^{DN}/+; *ey*-Flp5, *Act>y+*>Gal4, *UAS*-GFP/+; FRT 82B, *tub*-Gal80/FRT82B, *scrib*^{1}; (L) *UAS-bsk*^{DN}/+; *ey*-Flp5, *Act>y+*>Gal4, *UAS*-GFP/+; FRT 82B, *tub*-Gal80/FRT82B, *UAS*-Msn, *scrib*^{1}; (N) *ey*-Flp1/+; *Act>y+*>Gal4, *UAS*-GFP/*UAS-Ras*^{V12}; FRT 82B, *tub*-Gal80/FRT82B, *scrib*^{1}; (O) *ey*-Flp1/+; *Act>y+*>Gal4, *UAS*-GFP/*UAS-Ras*^{V12}; FRT 82B,

tub-Gal80/ FRT82B, *UAS*-Msn, *scrib*^{1}; (P) *ey*-Flp5, *Act>y+>*Gal4, *UAS*-GFP/*UAS-Ras*^{V12}; FRT 82B, *tub*-Gal80/FRT82B; (Q) *ey*-Flp5, *Act>y+>*Gal4, *UAS*-GFP/*UAS-Ras*^{V12}; FRT82B, *tub*-Gal80/ FRT82B, *UAS*-Msn.

Figure 4

(A and I) *nub*-Gal4/+; (B) *nub*-Gal4/+; *UAS*-Msn/+; (C) *nub*-Gal4/*UAS-ft-IR*; (D) *nub*-Gal4/*UAS-ft-IR*; *UAS*-Msn/+; (F) *ex-lacZ*/+; *hh*-Gal4/*UAS*-Msn; (G) *ex-lacZ*/*UAS-ft-IR*; *hh*-Gal4/+; (H) *ex-lacZ*/UAS-*ft-IR*; *hh*-Gal4/*UAS*-Msn; (J) *nub*-Gal4*/UAS*-Ft$^{\Delta ECD}$*;* (K) *nub*-Gal4/*UAS*-Ft$^{\Delta ECD}$*; wts*^{X1}*/+;* (L) *nub*-Gal4/*UAS*-Ft$^{\Delta ECD}$*; msn*^{3208}*/+;* (N) *en*-Gal4, *UAS*-GFP/+; *msn-lacZ*/+; (O) *en*-Gal4, *UAS*-GFP/*UAS-yki-IR*; *msn-lacZ*/+; (P) *en*-Gal4, *UAS*-GFP/*UAS-sd-IR*; *msn-lacZ*/+; (Q) *en*-Gal4, *UAS*-GFP/+; *msn-lacZ*/*UAS*-Yki; (R) *en*-Gal4, *UAS*-GFP/+; *msn-lacZ*/*UAS*-Yki^{S168A}; (S) *en*-Gal4, *UAS*-GFP/*UAS-sd-IR*; *msn-lacZ*/*UAS*-Yki^{S168A}.

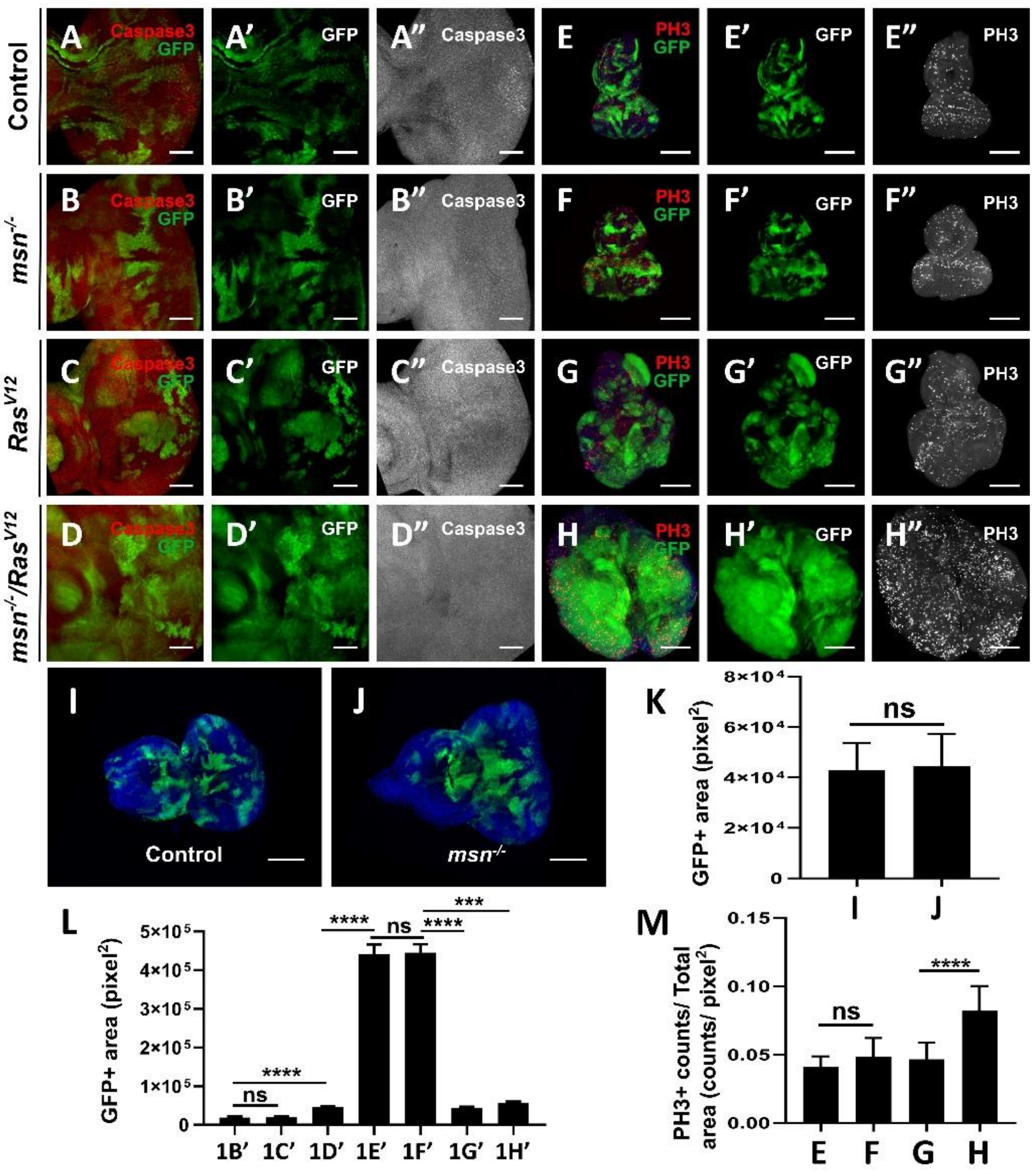

Figure S1. Loss of *msn* collaborates with *Ras*^{V12} to induce tumorigenesis

(A-H) Fluorescence micrographs of eye discs are shown. Loss of *msn* collaborates with *Ras*^{V12} to induce cell autonomous proliferation, marked by PH3 (E-H), but not apoptosis, marked by caspase3 (A-D). (I-K) Compared with control, loss of *msn* alone has no significant overgrowth (I-J). Quantification of GFP positive area in I-J (K). (L) Quantification of GFP positive area in Figure 1B'-H'. (M) Quantification of relative PH3 positive cells in E-H. ***P<0.001(mean + S.D.), ****P<0.0001(mean + S.D.). Scale bars represent 50 µm (A-D), 100 µm (E-J). Genotypes are as follows: (A, E and I) *ey*-Flp1/+; *Act*>*y*+> Gal4, *UAS*-GFP/+; *tub*-Gal80, FRT79E/FRT79E; (B, F and J) *ey*-Flp1/+; *Act*>*y*+>Gal4, *UAS*-GFP/ +; *tub*-Gal80, FRT79E/*msn*^{3208}, FRT79E; (C and G) *ey*-Flp1/+; *Act*>*y*+>Gal4, *UAS*-GFP/*UAS-Ras*^{V12}; *tub*-Gal80, FRT79E/FRT79E; (D and H) *ey*-Flp1/+; *Act*>*y*+>Gal4, *UAS*-GFP/*UAS-Ras*^{V12}; *tub*-Gal80, FRT79E/*msn*^{3208}, FRT79E.

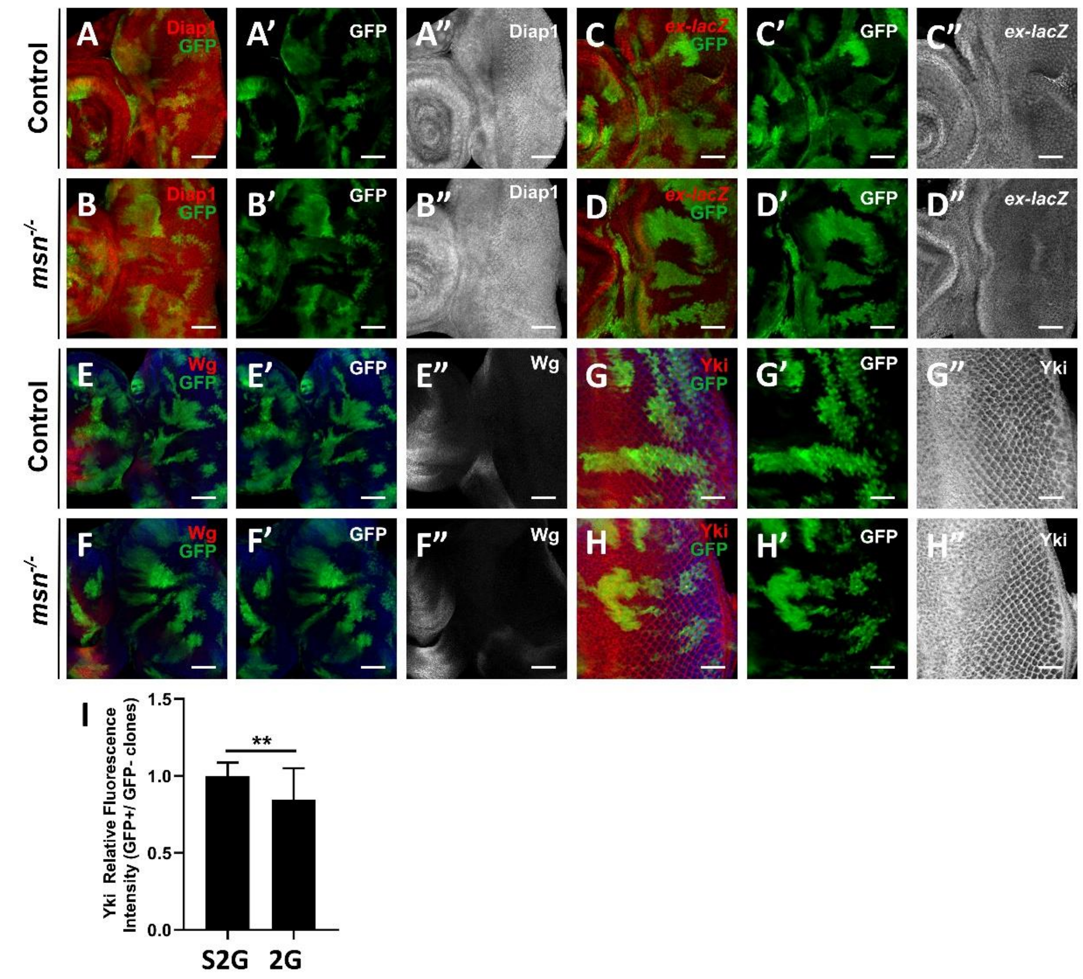

Figure S2. Loss of *msn* alone does not affect Hippo signaling activation

(A-H) Fluorescence micrographs of GFP-labeled clones of eye discs are shown. Compared with control, loss of *msn* alone has no obvious effect on Hippo target genes, including Diap1, Wg, and *ex*. Scale bars represent 50 μm (A-F), 20 μm (G-H). (I) Quantification of Yki relative fluorescence intensity in Figures S2G and 2G. ***P*<0.01 (mean + S.D.). Genotypes are as follows: (A, E and G) *ey*-Flp1/+; *Act*>*y*+>Gal4, *UAS*-GFP/+; *tub*-Gal80, FRT79E/FRT79E; (B, F and H) *ey*-Flp1/+; *Act*>*y*+>Gal4, *UAS*-GFP/+; *tub*-Gal80, FRT79E/*msn*^{3208}, FRT79E. (C) *ey*-Flp1/+; *Act*>*y*+>Gal4, *UAS*-GFP/*ex-lacZ*; *tub*-Gal80, FRT79E/ FRT79E; (D) *ey*-Flp1/+; *Act*>*y*+>Gal4, *UAS*-GFP/*ex-lacZ*; *tub*-Gal80, FRT79E/ *msn*^{3208}, FRT79E.

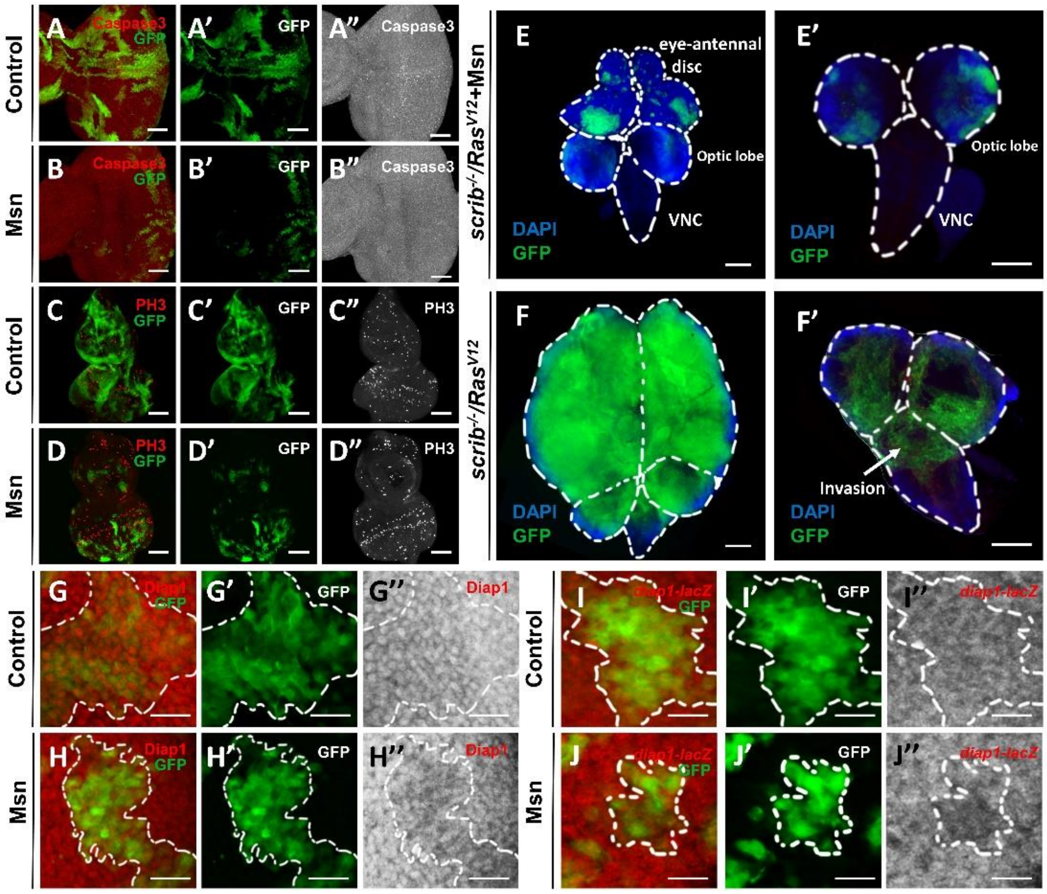

Figure S3. Msn overexpression suppresses tumorigenesis.

(A-D) Fluorescence micrographs of eye discs are shown. Compared with wild-type (A and C), Msn overexpression alone does not induce significant apoptosis (B) or proliferation (D), as indicated by Caspase 3 and PH3 staining, respectively. (E-F) Msn overexpression suppressed *scrib*$^{-/-}$/*Ras*^{V12} induced tumor overgrowth (E-F) and VNC invasion behavior (E'-F'). (G-J) Zoom in images of Figure 3C-F. Scale bars represent 50 μm (A-B), 100 μm (C-F), 20 μm (G-J). Genotypes are as follows: (A, C, G and I) *ey*-Flp5, *Act*>*y*+>Gal4, *UAS*-GFP/+; FRT82B, *tub*-Gal80/ FRT82B; (B, D, H and J) *ey*-Flp5, *Act*>*y*+>Gal4, *UAS*-GFP/+; FRT82B, *tub*-Gal80/ FRT82B, *UAS*-Msn; (E) *ey*-Flp1/+; *Act*>*y*+>Gal4, *UAS*-GFP/ *UAS*-*Ras*^{V12}; FRT82B, *tub*-Gal80/ FRT82B, *scrib*^{1}, *UAS*-Msn; (F) *ey*-Flp1/+; *Act*>*y*+>Gal4, *UAS*-GFP/ *UAS*-*Ras*^{V12}; FRT82B, *tub*-Gal80/ FRT82B, *scrib*^{1}.

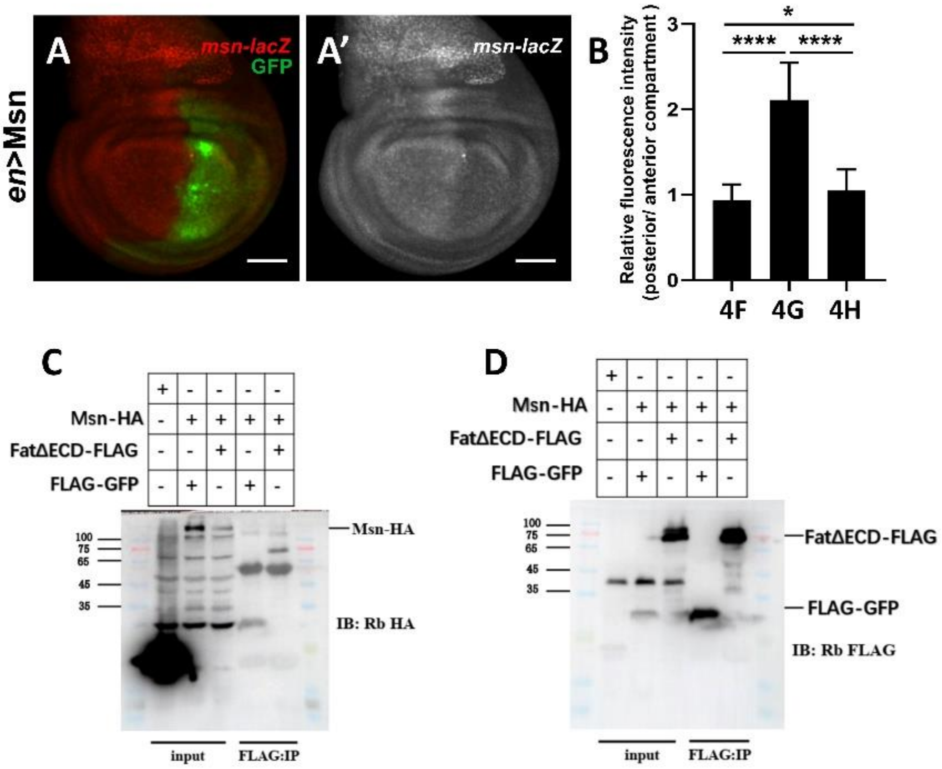

Figure S4. Msn regulates Hippo signaling in a feedback manner

(A-A') Fluorescence micrographs of wing discs are shown. Msn overexpression suppresses msn itself transcription. Scale bars represent 100 μm (A-A'). Genotypes are as follows: (A-A') *en*-Gal4, *UAS*-GFP/ +; *msn-lacZ*/*UAS*-Msn. (B) Quantification of Relative fluorescence intensity in Figure 4F-H. **P*<0.05(mean + S.D.), *****P*<0.0001(mean + S.D.). (C-D) Immunoprecipitation experiment indicated that no physical interaction between $Ft^{\Delta ECD}$ and Msn in S2 cells. Lysates expressing the indicated constructs were immunoprecipitated (IP) and probed with the indicated antibodies. Msn-HA was not detected in $Ft^{\Delta ECD}$-FLAG immunoprecipitation (C). Conversely, $Ft^{\Delta ECD}$-FLAG was detected in FLAG immunoprecipitation indicated $Ft^{\Delta ECD}$-FLAG plasmid has been transfected into S2 cells (D).

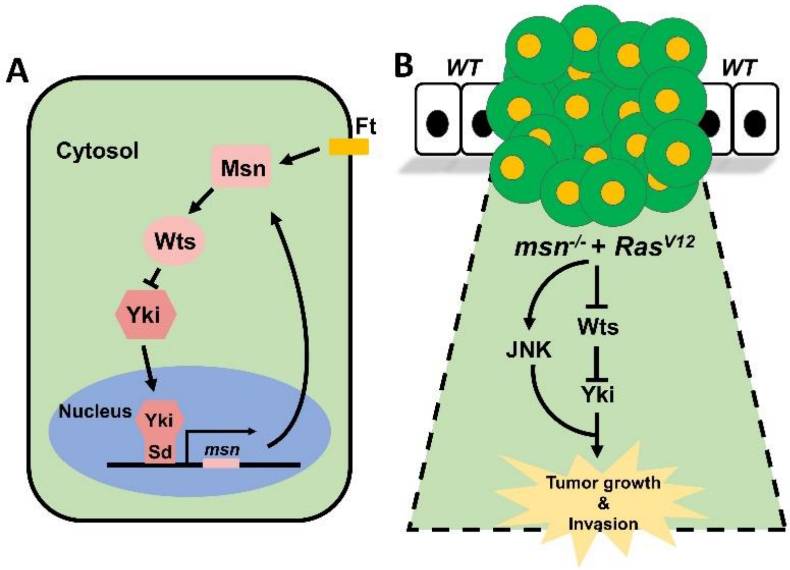

Figure S5. A schematic model depicting the role of Msn in regulating Hippo signaling and tumorigenesis.

(A) Msn acts as downstream of Ft to regulate Hippo pathway in a negative feedback manner. (B) Msn disruption cooperates with Ras^{V12} to drive tumorigenesis by inducing JNK pathway activation and Hippo pathway inactivation.
